# Effects of Oral Nutritional Supplementation on Body Composition and Bone Health in Undernourished Children: A Randomized Controlled Study

**DOI:** 10.3390/jcm14196972

**Published:** 2025-10-01

**Authors:** Anuradha Khadilkar, Arati Ranade, Neelambari Bhosale, Swati Hiremath, Nirali Mehta

**Affiliations:** 1 Jehangir Clinical Development Center Pvt. Ltd., Jehangir Hospital Premises, 32, Sassoon Road, Pune 411001, Maharashtra, India; arati.ranade@jcdc.co.in (A.R.); neelambari@jcdc.co.in (N.B.); swati.hiremath@jcdc.co.in (S.H.); 2 PHARMA-STATS, 207, Symmers, B/w Sarkhej-Santipura Cross Road, Sanand-Sarkhej Rd., Ahmedabad 382210, Gujarat, India; nirali.mehta@pharma-stats.com

**Keywords:** body composition, bone mineral content, bone mineral density, dual-energy X-ray absorptiometry, Indian children aged 3 to 6.9 years, lean mass, fat mass, oral nutritional supplement, picky eating, undernutrition

## Abstract

**Background/Objectives**: While oral nutritional supplements (ONSs) are known to support general growth in undernourished children, their specific effects on body composition and bone health remain underexplored. This manuscript evaluates the impact of ONS combined with dietary counselling (DC) on these outcomes in Indian children aged 3 to 6.9 years at nutritional risk, within the framework of a larger randomized controlled trial assessing multiple growth parameters. **Methods**: This prospective, randomized controlled trial was conducted among Indian children, both male and female participants with picky eating habits and at risk of undernutrition, aged 3 to 6.9 years (height-for-age and weight-for-height below the 25th percentile per WHO Growth Standards and Growth Reference). Participants were randomized in a 1:1 ratio to receive either ONS + DC or DC alone for 6 months. Body composition, bone mineral density (BMD), and bone mineral content (BMC) were measured using dual-energy X-ray absorptiometry (DXA) at baseline and after 6 months. Group differences were analyzed to evaluate intervention effects. **Results**: A total of 223 children were enrolled and randomized. At 6 months, the test group showed significantly greater improvements in BMD (0.023 g/cm^2^) compared to the control (0.017 g/cm^2^; *p* = 0.004), and a greater gain in BMC (36.60 g vs. 28.48 g; *p* =0.0007). Lean mass increased significantly more in the test group (926.33 g) versus the control (801.48 g; *p* = 0.0401). Fat mass showed a numerical reduction in the test group (−171.42 g) compared with the control group (−114.60 g), although this difference was not statistically significant. **Conclusions**: These findings highlight the potential of targeted nutritional interventions to favorably improve body composition and bone health during critical growth periods in undernourished children, offering a promising approach to address early-life nutritional deficits with lasting health benefits.

## 1. Introduction

Childhood represents a critical window of opportunity for growth, neurodevelopment, and the establishment of lifelong health trajectories. Adequate nutrition during this period is fundamental not only for supporting linear growth and appropriate weight gain but also for promoting bone health and overall physical growth, as evidenced by Saavedra and Prentice [1] and Prentice et al. [2].

Undernutrition, including stunting, wasting, and underweight, remains a major global public health challenge with far-reaching consequences for child survival, health, and developmental potential, as underscored in the UNICEF, WHO, and World Bank Joint Malnutrition Estimates [3]. As of 2024, the global estimates report that 150.2 million children under five years were stunted, 42.8 million were wasted, and 35.5 million were overweight, reflecting a persistent double burden of malnutrition worldwide [3]. Despite decades of public health initiatives, India continues to struggle with widespread undernutrition. According to the National Family Health Survey-5, 36% of children under five are stunted, 19% are wasted, and 32% are underweight [4].

The consequences of undernutrition extend beyond impaired linear growth and inadequate weight gain, profoundly affecting body composition and bone accretion [5]. Wells et al. highlighted that undernourished children typically present with reduced lean body mass, altered fat distribution, and impaired bone mineralization, all of which compromise physical function, immune competence, neurocognitive outcomes, and long-term cardiometabolic health [5]. Body composition, defined by the amount of fat mass and lean mass, and bone health, assessed through bone mineral content (BMC) and bone mineral density (BMD), are now widely recognized as critical markers of growth quality and predictors of future health outcomes [6,7].

A key driver of these deficits is the widespread lack of essential macronutrients and micronutrients [2], particularly protein, calcium, vitamin D, vitamin C, and vitamin K. Golden’s proposed nutrient requirements emphasize the need to address both macronutrient and micronutrient insufficiencies in undernourished populations [8]. Vitamin D deficiency impairs calcium absorption, heightening the risk of rickets, while vitamin C is essential for collagen synthesis in bone matrix and vitamin K plays a key role in regulating bone mineralization and turnover [9,10,11]. Protein-energy malnutrition leads to muscle wasting, reducing mechanical loading on bone and impairing skeletal development, as described by Mehta et al. [12]. Without timely intervention, these nutritional deficiencies heighten the risk of fractures, physical disability, and chronic diseases later in life [13].

Formulated food supplements and oral nutritional supplements (ONSs) have emerged as promising interventions to address undernutrition. Fabiansen et al. demonstrated that formulated food supplements effectively increase fat-free mass in children with moderate acute malnutrition [14]. More recently, ONSs, providing a concentrated source of energy, high-quality protein, and critical micronutrients that support catch-up growth, have been shown to improve bone health and body composition. The randomized controlled trial by Ow et al. shows that ONS not only promote weight gain but also enhance linear growth and skeletal outcomes, indicating meaningful recovery beyond simple weight restoration [15]. As emphasized by Golden, nutrient-dense interventions are essential to address both energy and micronutrient deficits and are critical components of effective nutritional rehabilitation strategies in undernourished children [8].

Given the high global burden of undernutrition and its long-term consequences, there is an urgent need for rigorously designed studies to evaluate the effects of ONS on body composition and bone health outcomes. This manuscript focuses on assessing the outcomes of ONS combined with dietary counseling in undernourished children under seven years of age, using dual-energy X-ray absorptiometry (DXA), the gold standard for pediatric body composition and bone assessment [16]. By addressing this critical evidence gap, this study aimed to inform evidence-based nutritional rehabilitation strategies and provide actionable insights to optimize the growth, health, and developmental trajectories of undernourished children.

## 2. Materials and Methods

### 2.1. Study Design and Participants

This manuscript presents findings on body composition and bone health outcomes from a previously reported prospective, randomized, controlled, open-label trial by Khadilkar et al. [17]. This trial evaluated the effect of ONS combined with dietary counselling (DC) on growth outcomes in Indian children aged 3.0 to 6.9 years who were at nutritional risk, with anthropometric results reported separately [17].

Participants were randomly assigned to receive either ONS plus DC (intervention group) or DC alone (control group), with a balanced ratio of male and female participants maintained across both groups. Study outcomes were evaluated at 3 and 6 months after the initiation of the intervention. In addition to growth parameters, detailed assessments of body composition and bone health were performed at baseline and end of study using DXA scan to provide a broader understanding of the physiological impact of the nutritional intervention.

This study was conducted between August 2023 and May 2024 at the Jehangir Clinical Development Centre Pvt. Ltd. in Pune, India, enrolling participants from two nearby primary schools. Eligible participants were children aged 3.0 to 6.9 years who met the criteria for nutritional risk, defined as having both height-for-age and weight-for-height below the 25th percentile according to the WHO Growth Standards and Growth Reference [18,19]. For children above 5 years, BMI-for-age below the 25th percentile was used as the eligibility criterion in place of weight-for-height percentiles, owing to the unavailability of WHO reference charts for this age group. Additional inclusion criteria included the ability to consume food and beverages orally, picky eating behavior characterized by strong food preferences, limited food variety, refusal to eat vegetables and/or foods from other food groups, unwillingness to try new foods, disruptive mealtime and aversions, and a habitual intake of milk. Children were excluded if they had known lactose intolerance, galactosemia, hypersensitivity to any component of the study product, developmental disabilities or physical disorders likely to affect growth (such as cerebral palsy or developmental delay), an immunocompromised state, or required a medically prescribed special diet.

The study protocol was reviewed and approved by the Institutional Ethics Committee of Jehangir Clinical Development Centre Pvt. Ltd. and registered with the Clinical Trials Registry of India (CTRI/2023/04/051566). The trial was conducted in accordance with the International Council for Harmonization of Technical Requirements for Pharmaceuticals for Human Use (ICH) E6(R2) guidelines for Good Clinical Practice (GCP), the Indian GCP guidelines, the Indian Council of Medical Research (ICMR) Ethical Guidelines (2017), and the ethical principles outlined in the Declaration of Helsinki.

### 2.2. Intervention

Participants in the intervention arm (subjects receiving the study product ONS, two servings per day along with dietary counselling) received a reconstituted nutritional supplement (PediaSure; Abbott Healthcare Private Limited, Mumbai, India), (203 g/L; 45.5 g ONS powder + 190 mL water per serving). The parents or Legal Authorized Representative (LAR) of participants in the interventional arm were trained during the first visit on the prescribed dosage, method of preparation, and timing of administration of the study product ONS.

The oral nutritional supplement used in this study was formulated to provide a balanced profile of macronutrients and micronutrients essential for catch-up growth. At standard dilution, each 100 mL delivered 91 kcal of energy, 2.85 g of protein, 3.03 g of fat, and 13.10 g of carbohydrates, and a wide range of vitamins and minerals. The carbohydrate component was derived from multiple sources, including skim milk powder, lactose, maltodextrin, sucrose, and fructo-oligosaccharides (FOS, 1.9%). The formulation was also enriched with functional nutrients such as linoleic acid, alpha-linolenic acid, and prebiotic FOS, supporting energy metabolism and gut health, and nutrients to support growth such as arginine, Vitamin K2 and casein phosphopeptides.

Dietary counseling was provided to subjects in both arms on the same day, tailored to their age, physical status, and daily physical activities. The dietary advice, delivered by the study center nutritionist, included a discussion of the subject’s regular diet and an explanation of the components of a healthy, balanced diet covering macronutrients such as carbohydrates, proteins, and fats, and micronutrients such as vitamins and minerals. The importance and function of each component were explained, along with how these nutrients could be incorporated into the subject’s daily meals. The parents or LARs of the participants were provided, in written or printed form, with a list of locally available foods that could be incorporated into the child’s daily diet, together with guidance on combining different food items to achieve a healthy and balanced diet. The session was intended to deliver brief training to the parents/LARs on age- and physical status–appropriate quality and quantity of dietary intake. Caregivers were advised on practical ways to support healthy eating at home, such as preparing nutritious meals and encouraging their child to consume a balanced diet based on the instructions provided. Participants were also instructed to maintain a structured subject diary, to record compliance to the study product and their daily food intake throughout the study period.

### 2.3. Study Procedure

Children within the target age group were enrolled from local schools and screened for eligibility during a dedicated screening visit. Upon obtaining written informed consent from parents/guardians and confirming eligibility, participants were randomized in a 1:1 ratio to either the intervention or control arm using the randomizeR package (v 3.0.1) in R (v 4.3.0, 64-bit). This study included four scheduled visits over a six-month intervention period: Visit 1 (screening and baseline), Visit 2 (1 month), Visit 3 (3 months), and Visit 4 (end-of-study at 6 months).

Anthropometric measurements were performed and are published in another paper [18] Bone composition and mineralization were assessed at baseline and at the end of the six-month intervention (Visits 1 and 4) via dual-energy X-ray absorptiometry (DXA), performed by a skilled technician. The scans evaluated BMD, BMC, lean mass, fat mass, and bone area. Nutrient intake was recorded at every visit using structured 24 h dietary recalls administered by trained personnel. Throughout this study, parents maintained daily logs documenting supplement adherence, dietary intake, and illness episodes. Adverse events were monitored continuously from enrollment through study completion.

### 2.4. Dual Energy X-Ray Absorptiometry

All participants underwent bone assessments using a GE Lunar iDXA scanner (GE Healthcare, Madison, WI, USA) equipped with a fan-beam and 64-channel detector (Software version: enCORE 16). The anatomical region scanned included the whole body, excluding the skull. Key parameters measured included bone mineral content (BMC, g), bone mineral density (BMD, g/cm^2^), and bone area (cm^2^). Total body BMD Z-scores obtained in the reports were based on US NHANES reference data. In addition to skeletal metrics, body composition data including lean mass, fat mass, and fat percentage were obtained. In this study, lean mass as reported by the GE Lunar iDXA is defined as all non-bone, non-fat tissue.

The cranium was excluded from total body analyses to avoid overestimation of bone mass, as it represents a disproportionately large and relatively stable region that may obscure growth-related changes in other skeletal sites [20]. Standardized procedures were followed to minimize motion artifacts during scanning, including the use of child-appropriate positioning aids and trained personnel to ensure compliance.

Intra-individual variability in DXA measurements at our study site has been previously assessed through cross-calibration in 31 children (14 boys, 17 girls; mean age 11.6 ± 4.0 years). The coefficients of variation (CV) were 0.8% for total body BMC, 0.7% for BMD, 0.7% for lean mass, and 1.0% for fat mass, as reported by Dongare-Bhor and colleagues in Bone [21]. These values indicate high reproducibility of the DXA system and provide confidence that the observed changes exceeded measurement error.

### 2.5. Sample Size and Statistical Analysis

The sample size was calculated based on a mean difference of 0.32 units between Group 1 (mean ≈ −3.00) and Group 2 (mean ≈ −2.68), with an estimated standard deviation of 0.76 derived from Lopriore et al. [22]. Using a two-sample *t*-test with 80% power and a two-sided significance level of 0.05, a total of 90 participants per group was determined to be sufficient. To accommodate an anticipated 30% attrition, the final sample size was increased to 117 participants per group, yielding a total of 234 participants.

Subsequent statistical analyses were performed using R software (version 4.3.1 or higher). Continuous variables were summarized using means and standard deviations, while categorical variables were described as frequencies and percentages. Changes from baseline (CFB) for BMD, BMC, and body composition parameters were analyzed using analysis of covariance (ANCOVA) models. The model included randomized treatment along with study centre, gender treatment*gender interaction, age (numeric), and baseline value as covariates. Treatment effects were expressed as least squares mean differences with corresponding two-sided 95% confidence intervals and *p*-values. Statistical significance was set at a two-sided alpha level of 0.05.

## 3. Results

### 3.1. Subject Disposition

**Figure 1:**
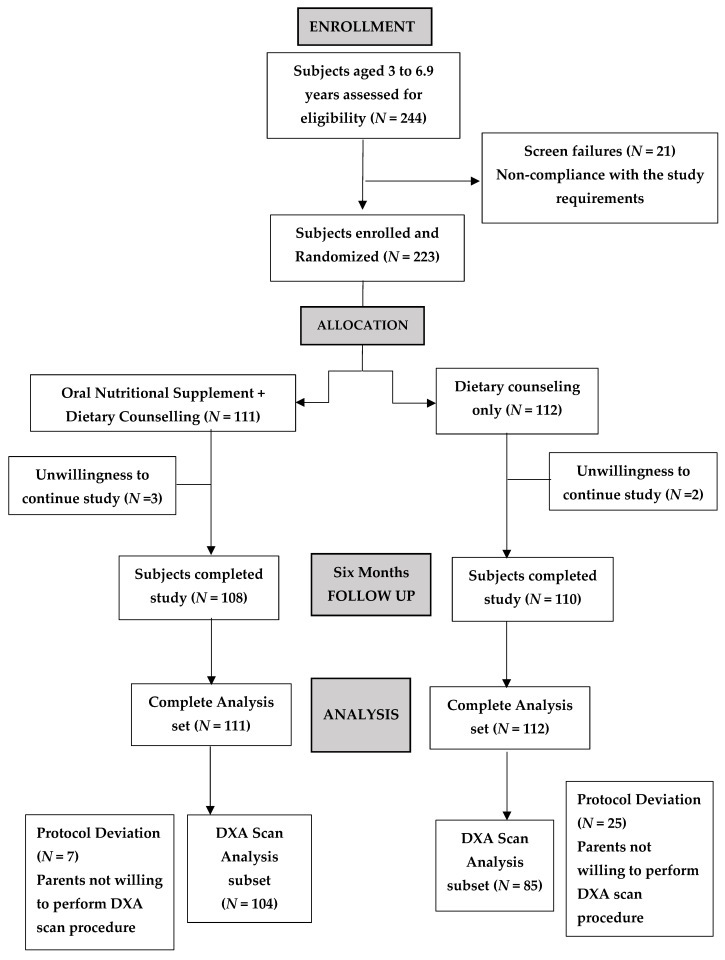
CONSORT Study flow diagram, Subject Disposition.

Comprehensive DXA-based assessments of body composition and bone health were performed, revealing key findings. Of the 244 subjects, screened, 21 were considered screen failure, resulting in enrollment of 223 participants, who were included in the final safety analysis. Three participants from the intervention arm (*N* = 111) and two participants from the DC arm (N = 112) discontinued before study completion. As a result, 218 participants completed this study and were included in the final efficacy analysis (Figure 1). For the purpose of bone assessments using DXA, data for 104 participants from the intervention arm and 85 participants from the DC arm was available for both baseline and 6 months visit. As 29 participant’s parents showed unwillingness to perform DXA and this non-compliance was reported as protocol deviations.

### 3.2. Baseline Demography

**Figure 2:**
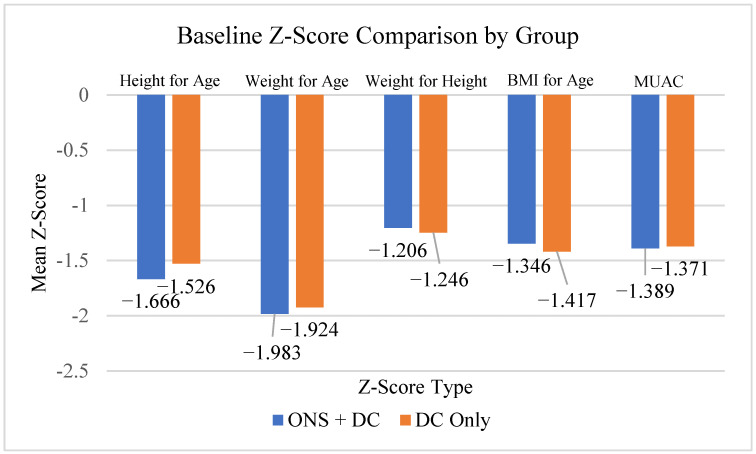
Comparison of Baseline Z-Score Types Between Groups.

The demographic and baseline characteristics of the study population are summarized in Table 1. A total of 223 children were enrolled, with 111 in the ONS plus dietary counseling group and 112 in the dietary counseling alone group. The gender distribution was balanced (50.2% male, 49.8% female), and the mean age across groups was 4.8 years. As shown in Table 1, baseline anthropometric parameters including mean height (101.3 cm), body weight (13.9 kg), and BMI (13.5 kg/m^2^) were comparable between groups. The mean Z-scores for height-for-age (−1.60 ± 0.5) and weight-for-age (−1.95 ± 0.5) indicated that participants were below the median of the WHO Growth Standards and Growth Reference, consistent with the defined nutritional risk. Mid-upper arm circumference Z-scores (mean: −1.38 ± 0.5) were also similar between groups. An intergroup comparison of baseline Z-score categories is illustrated in Figure 2.

As reported in a separate published manuscript [17], children in the intervention group demonstrated greater linear growth, providing context for the present findings on body composition and bone health; comprehensive anthropometric outcomes are presented in that publication.

### 3.3. Bone Health and Body Composition

As described below, Table 2 provides a summary of changes in bone health and body composition parameters after 6-month intervention across the study groups. At six months, both intervention and control arms demonstrated improvements in skeletal and body composition parameters, with consistently greater mean changes observed in the ONS + DC group compared to DC group alone.

The increase in BMD from baseline was more pronounced in the ONS + DC group (mean change: 0.023 g/cm^2^) relative to the DC group (0.017 g/cm^2^). The least-squares (LS) mean difference was 0.007 g/cm^2^ (95% CI: 0.002, 0.011), achieving statistical significance (*p* = 0.0040). Bone mineral content (BMC) followed a similar pattern, with the ONS + DC group exhibiting a greater mean increase (36.595 g) compared to the DC group (28.483 g). The LS mean difference of 8.34 g (95% CI: 3.58, 13.10) was statistically significant (*p* = 0.0007), indicating a more robust gain in bone mass with the combined intervention.

The mean increase in bone area was also higher in the ONS + DC group (50.622 cm^2^) than in the DC group (42.287 cm^2^). Although the LS mean difference of 6.97 cm^2^ (95% CI: −1.00, 14.94) did not reach statistical significance (*p* = 0.0862), the observed trend favored the ONS + DC group.

Total lean mass improved in both groups, with a greater mean increase in the ONS + DC group (926.331 g) relative to the DC group (801.482 g). The LS mean difference of 135.14 g (95% CI: 6.18, 264.11) was statistically significant (*p* = 0.0401), supporting a beneficial effect on lean tissue accretion associated with ONS supplementation.

Both groups exhibited reductions in total fat mass from baseline. The ONS + DC group demonstrated a greater mean decrease (−171.418 g) than the DC group (−114.597 g), though the LS mean difference (−57.95 g; 95% CI: −192.38, 76.48) did not attain statistical significance (*p* = 0.3961). A similar pattern was observed for fat percentage, with a greater reduction in the ONS + DC group (−2.546%) compared to the DC group (−2.134%); the LS mean difference of −0.52% (95% CI: −1.18, 0.14) was not statistically significant (*p* = 0.1211).

Total Body BMD Z-scores remained largely unchanged in both groups. The LS mean difference was 0.00 (95% CI: −0.10, 0.11; *p* = 0.9715), indicating comparable effects of the interventions on this parameter. Overall, these findings capture the intervention’s effects on body composition and bone health at the 6-month endpoint.

Lean Mass Index (LMI) and Fat Mass Index (FMI) were evaluated alongside skeletal outcomes. At baseline, both groups showed comparable values for LMI and FMI. Over 6 months, the ONS + DC group exhibited a small decline in LMI, while the DC group demonstrated a modest increase. In contrast, FMI decreased in both groups, with a greater reduction observed in the ONS + DC group. Between-group differences for changes in both indices were statistically significant as presented in Table 2.

## 4. Discussion

### 4.1. Key Findings

Addressing childhood undernutrition requires interventions that go beyond caloric repletion to support functional and structural growth. In this context, our study findings stemming from a six-month intervention combining ONS with dietary counselling underscore the critical role of early, targeted nutritional approaches in promoting high-quality growth, characterized by lean mass accretion, optimal bone mineralization, and linear growth improvement, while mitigating the risk of excess fat accumulation [1,23].

The capacity of ONS to induce multidimensional improvements in growth parameters is further supported by mechanistic modelling. Chheda et al. employed an in silico system physiology approach to predict outcomes in preschool-aged children, demonstrating a 1.7-fold enhancement in weight gain velocity and a 1.04-fold increase in linear growth with ONS compared to standard diets. The model used ordinary differential equations to simulate nutrient intake, energy balance, and tissue-specific growth including fat, lean, and bone mass based on established physiological parameters. These simulated gains were accompanied by increases in lean mass, muscle mass, and bone mass, providing a mechanistic rationale for the qualitative growth patterns observed in our cohort [24].

Extending these insights, our data reveal that the ONS + DC group experienced marked improvements in BMD and BMC, reinforcing the biological plausibility and effectiveness of a comprehensive nutrient-dense intervention for bone accretion. The intervention group’s superior response may be attributed to targeted provision of calcium, vitamin D, and high-quality protein. These nutrients are essential for hydroxyapatite crystallization and collagen matrix formation [7,25], thereby supporting both trabecular and cortical bone growth during this critical window [26,27]. Additionally, the presence of arginine, vitamin K2, and casein phosphopeptides (CPPs) may have contributed to measurable enhancements in lean mass, BMC, and BMD. Arginine is known to stimulate anabolic signaling through growth hormone pathways; vitamin K2 facilitates osteocalcin activation and calcium utilization; and CPPs enhance the bioavailability of calcium and zinc by stabilizing them in soluble complexes, all contributing synergistically to skeletal maturation [15,28,29].

The findings from the current study in Indian children corroborate and extend those reported in the recently published SPROUT trial conducted in Vietnam by Ow et al., which evaluated the effects of long-term ONS combined with dietary counseling on growth, body composition, and bone mineralization. Although the SPROUT study involved a longer intervention period, our 6-month trial also demonstrated significant improvements in body composition and bone health parameters, highlighting the early benefits of this nutritional strategy in children at risk for undernutrition. Notably, our study contributes new evidence from a distinct regional cohort of Indian children, who are known to exhibit the “thin-fat” phenotype, a pattern described by Khadilkar et al. [30], and further supported by the work of Lakshmi et al. [31], and Nakavachara et al. [32]. These studies show that Indian children tend to have relatively higher body fat but lower lean mass and BM for a given BMI when compared with Western and Southeast Asian peers. These population-specific body composition traits likely influenced both the baseline DXA profiles and the observed response to the intervention in Indian children. While the overall direction of effect aligns with the SPROUT trial, the present findings offer population-specific insights into how Indian children respond to ONS plus dietary counseling, thereby supporting the development of regionally tailored strategies for improving pediatric body composition and bone health outcomes.

Also, Shatrugna et al. documented increased fat-free mass, BMC, and BMD in undernourished Indian schoolchildren following micronutrient supplementation achieved without promoting excess adiposity, demonstrating the feasibility of nutrient-efficient, functional growth [33]. These population-specific body composition traits likely influenced both the baseline DXA profiles and the observed response to the intervention in Indian children.

Given the integral role of the skeleton in linear growth, the height gains observed in our study likely reflect not only increased stature but also concurrent improvements in bone mass and structure, which are critical components of bone health during childhood and adolescence [17,34]. This bone growth is essential for achieving peak bone mass, which is vital for strong bones and reducing the risk of fractures later in life [35]. Together, these parallel advances highlight the long-term value of early nutritional intervention in supporting both optimal height attainment and skeletal development in undernourished populations.

Complementing improvements in density and structure, geometric adaptations provide further insight into bone growth trajectories. The trends toward increased bone area in the ONS + DC group point to potential changes in skeletal geometry. Although the intergroup difference in bone area did not achieve statistical significance, the upward trajectory is noteworthy. Bone area expansion, largely driven by periosteal growth, increases surface area for mineral deposition and enhances structural strength via cortical thickening [36]. Even modest geometric adaptations confer meaningful gains in structural integrity, particularly in the context of pediatric bone development [37].

Parallel increases in total lean mass further affirm the skeletal benefits of the intervention. Lean mass is a key determinant of functional growth, metabolic resilience, and physical performance [38]. In this study, children receiving ONS + DC achieved significantly greater gains in lean mass, likely due to enhanced protein intake and the synergistic actions of micronutrients on bone–muscle crosstalk. Mechanical loading from muscle activity is a known osteogenic stimulus, and the coordinated growth of muscle and bone highlights the potential of comprehensive nutritional interventions to support the integrated growth of the skeletal unit [39,40]. These findings are consistent with previous research showing that lean tissue development contributes not only to structural competence but also to enhanced metabolic health and reduced morbidity risk in children [41]. This relationship is further supported by a large meta-analysis by Deng et al. [42], which included over 21,000 children and adolescents and demonstrated a strong, independent association between lean mass and bone mineral density across multiple skeletal sites. The findings highlight the role of muscle accrual in promoting functional skeletal development and showed a stronger association in Asian populations, consistent with the patterns observed in our Indian cohort. Collectively, these results underscore the consistent link between lean mass and bone mineral density in children.

In addition to absolute measures, indices normalized for height were also examined. In the ONS + DC group, both absolute lean mass and fat mass increased over the 6-month period. However, due to a significant gain in height (mean height-for-age z-score increase of +0.6, indicating linear catch-up growth), LMI and FMI declined. In contrast, the DC-only group showed no evidence of linear catch-up growth (mean HAZ change of −0.09) but exhibited a modest increase in LMI. These patterns suggest that in the ONS + DC group, linear growth was prioritized during nutritional recovery, with gains in stature outpacing proportional increases in body mass. Despite LMI and FMI decreases, absolute lean mass increased, and fat mass values decreased, consistent with positive body composition changes. These findings are aligned with evidence from previous research, which identifies lean mass as a significant determinant of BMD [43] and highlights the importance of considering proportional metrics like LMI during growth phases. The increase in TLM supports the role of lean mass in skeletal health, even as changes in LMI reflect the dynamic interplay between growth and body composition [40,43].

To further contextualize these shifts, we examined Total Body BMD Z scores as an indicator of skeletal health. Z-scores could only be computed for a subset of participants, as reference data were available only for those older than 5 years. The inclusion of Total Body Z-scores allowed for a standardized assessment of skeletal status in this population. Baseline values confirmed suboptimal bone mineralization in undernourished children, reflecting the broader skeletal impact of chronic nutritional deficits. However, an interpretive challenge is that the NHANES reference data used for Z-score calculation are based on a U.S. population, which may not be directly representative of Indian children due to ethnic, nutritional, and developmental differences. Prior studies, of Khadilkar et al. [31], have shown that healthy Indian children exhibit lower BMD and lean mass compared to Western norms, a trend attributed in part to the ‘thin-fat’ phenotype. Therefore, while NHANES-based Z-scores provide a consistent and internationally recognized benchmark, they may underestimate normative bone values in Indian children and overstate the prevalence of low BMD. To address this, we interpreted Z-scores with caution, avoided sole reliance on absolute diagnostic cut-offs [44], and emphasized within-group and longitudinal comparisons, which are more appropriate for evaluating intervention effects in ethnically distinct populations.

Over the six-month period, Total Body BMD Z-scores remained stable in both groups, with minimal changes observed in the ONS + DC group from baseline. Although the between-group difference was not statistically significant, the directional trend suggests a potential protective effect of supplementation on skeletal integrity. Interestingly, despite significant changes in body composition and skeletal parameters, overall Total Body Z-scores were maintained throughout the intervention. In a nutritionally vulnerable population, such stability likely reflects the preservation of normative bone health, which is a clinically meaningful outcome. However, Total Body BMD Z-scores may lack the sensitivity to detect regional or microarchitectural skeletal changes, underscoring the need for more granular imaging and extended follow-up [45,46]. These findings highlight the importance of including skeletal endpoints in future nutritional interventions targeting undernourished populations.

Taken together, these findings affirm the multidimensional benefits of ONS when integrated with dietary counselling in undernourished children. Beyond significant improvements in height and weight [17], the intervention promoted lean mass accrual, reductions in adiposity, BMD Z-scores, and maintenance of age-appropriate bone mineralization. These outcomes highlight the synergistic effects of energy and nutrient-dense supplementation on growth and skeletal growth. Notably, the favorable shift toward increased lean mass without excess fat gain reflects an enhancement in growth quality. These results are relevant for informing nutritional practices in settings where undernutrition and suboptimal growth remain prevalent concerns.

### 4.2. Strengths and Limitations

A key strength of this study is its randomized controlled design, which strengthens internal validity and supports robust conclusions on the effects of ONS combined with dietary counselling on body composition and bone health in undernourished children aged 3–6.9 years. Targeting a nutritionally at-risk pediatric group identified through standardized anthropometric and clinical criteria enhances real-world relevance, as early intervention during this critical growth phase may help prevent long-term skeletal and developmental deficits. The use of DXA, the gold standard for pediatric body composition assessment, ensured precise, reproducible measurements of BMC, BMD, lean mass, fat mass and Z-scores. This comprehensive approach provides a clear picture of skeletal and body composition growth responses. Rigorously standardized protocols further enhanced reliability. Notably, the six-month duration offers meaningful early insights into the benefits of nutritional support on growth trajectories.

Several limitations should be noted. The six-month duration may not reflect long-term skeletal outcomes, and findings may not generalize across diverse populations. DXA results can be affected by bone size, development, and movement. Total Body BMD Z-scores were limited to children ≥5 years. iDXA GE Lunar uses extensive reference data including USA and Northern European subjects, as well as NHANES, and numerous regional databases, which may not fully represent this cohort [47,48]. Bone-related biochemistry and fracture history were not assessed. Socioeconomic data were not collected; however, this is not expected to affect the main findings, which focus on objective measures of body composition and bone health rather than affordability. Finally, the lack of physical activity data remains a relevant limitation that may confound outcome interpretation. Nonetheless, this study provides encouraging early evidence of nutritional benefits on bone health and body composition.

### 4.3. Future Prospectives

Future studies should use a combination of imaging methods, longer follow-up periods, and detailed monitoring of dietary intake to better understand the lasting effects of oral nutritional supplementation on bone and overall growth. Additionally, assessing functional outcomes like physical performance and fracture risk would offer a more complete picture of how nutritional interventions benefit children during key growth stages.

## 5. Conclusions

This investigation demonstrates that a combined approach of oral nutritional supplementation and dietary counselling induces early and meaningful enhancements in body composition and bone mineralization among nutritionally vulnerable children aged 3 to 6.9 years. Specifically, the intervention led to increased lean mass without a corresponding gain in fat mass percentage, indicating a shift toward a healthier and more optimized body composition. Concurrently, bone health improved, as evidenced by significant increases in BMC and BMD. By promoting both bone mineral accrual and favorable body composition, this intervention addresses critical developmental windows where nutritional deficits could otherwise compromise lifelong skeletal health. The results underscore the necessity of integrating multifaceted nutritional approaches tailored to children at risk, thereby moving beyond traditional single-nutrient approaches. Future research should prioritize longitudinal analyses and functional outcomes to elucidate the sustained clinical benefits and inform scalable public health interventions aimed at breaking the cycle of childhood undernutrition and its sequelae.

## Figures and Tables

**Table 1:** Demographic and Baseline Characteristics of Study Participants by Group.

Parameters		Statistics	ONS + Dietary Counselling *(N* = 111)	Dietary Counselling (*N* = 112)	Total *(N* = 223)
**Gender**	**Male**	*n* (%)	59 (53.2)	53 (47.3)	112 (50.2)
	**Female**	*n* (%)	52 (46.8)	59 (52.7)	111 (49.8)
**Age (Years)**		Mean (SD)	4.8 (0.9)	4.8 (0.9)	4.86 (0.9)
**Average Height (cm)**		Mean (SD)	101.0 (5.8)	101.7 (5.6)	101.3 (5.7)
**Average Body Weight (kg)**		Mean (SD)	13.9 (1.3)	14.0 (1.3)	13.9(1.3)
**BMI (kg/m^2^)**		Mean (SD)	13.6 (0.5)	13.5 (0.6)	13.5 (0.5)

Abbreviations: *N* = Total number of subjects in treatment group, *n* = Total number of non-missing subjects in specified category, SD = Standard Deviation.

**Table 2:** Changes in Body Composition and Bone Health Parameters from Baseline to 6 Months by Study Groups.

Parameters Visits	ONS + Dietary Counselling (*N* = 111)		LS Mean Difference [95% CI]	*p*-Value	Dietary Counselling (*N* = 112)	
	***n***	**Mean (SD)**			***n***	**Mean (SD)**
**BMD (g/cm^2^)**						
Baseline	111	0.424 (0.0417)			112	0.424 (0.0436)
Visit 4 (6 Months)	104	0.445 (0.0422)			85	0.434 (0.0437)
CFB of BMD (g/cm^2^) Visit 4 (6 Months)	104	0.023 (0.0168)	0.007 [0.002, 0.011]	0.0040 *	85	0.017 (0.0138)
**BMC (g)**						
Baseline	111	294.051 (62.4857)			112	299.647 (61.5589)
Visit 4 (6 Months)	104	328.338 (65.1734)			85	321.561 (64.7891)
CFB of BMC (g)Visit 4 (6 Months)	104	36.595 (18.9604)	8.34 [3.58, 13.10]	0.0007 *	85	28.483 (12.7077)
**Area (cm^2^)**						
Baseline	111	680.091 (89.9563)			112	693.557 (88.0579)
Visit 4 (6 Months)	104	728.365 (90.0855)			85	730.937 (88.7503)
CFB of Area (cm^2^) Visit 4 (6 Months)	104	50.622 (32.3477)	6.97 [−1.00, 14.94]	0.0862	85	42.287 (21.5326)
**Total Fat (g)**						
Baseline	111	3852.883 (838.1879)			112	3994.836 (1040.1678)
Visit 4 (6 Months)	104	3683.358 (899.4342)			85	3935.513 (1121.9666)
CFB of Total Fat (g) Visit 4 (6 Months)	104	−171.418 (454.2310)	−57.95 [−192.38, 76.48]	0.3961	85	−114.597 (471.8246)
**Total Fat (%)**						
Baseline	111	26.627 (3.8024)			112	27.141 (4.9222)
Visit 4 (6 Months)	104	24.183 (4.2023)			85	25.442 (4.7672)
CFB of Total Fat (%) Visit 4 (6 Months)	104	−2.546 (2.3283)	−0.52 [−1.18, 0.14]	0.1211	85	−2.134 (2.3610)
**Total Lean Mass (g)**						
Baseline	111	10,090.978 (1453.6623)			112	10,117.221 (1298.8145)
Visit 4 (6 Months)	104	10,979.006 (1571.6173)			85	10,861.615 (1480.8834)
CFB of Total Lean Mass (g) Visit 4 (6 Months)	104	926.331 (513.2353)	135.14 [6.18, 264.11]	0.0401 *	85	801.482 (358.7196)
**Total Body BMD ^$^ Z-score (+/−)**						
Baseline	52	−1.523 (0.7944)			51	−1.434 (0.7491)
Visit 4 (6 Months)	61	−1.479 (0.7321)			50	−1.463 (0.7054)
CFB of Total Body Z-score (+/−) Visit 4 (6 Months)	47	−0.014 (0.2081)	0.00 [−0.10, 0.11]	0.9715	34	−0.044 (0.2995)
**LMI (kg/m^2^)**						
Baseline	111	9.861 (0.8788)			112	9.764 (0.8256)
Visit 4 (6 Months)	104	9.590 (0.7809)			85	10.067 (0.8040)
CFB of Lean Mass Index (kg/m^2^)	104	−0.257 (0.6028)	−0.53 [−0.67, −0.39]	<0.0001	85	0.293 (0.4014)
**FMI (kg/m^2^)**						
Baseline	111	3.775 (0.7210)			112	3.862 (0.9271)
Visit 4 (6 Months)	104	3.231 (0.7258)			85	3.650 (0.9454)
CFB of Fat Mass Index (kg/m^2^)	104	−0.557 (0.3795)	−0.28 [−0.40, −0.16]	<0.0001	85	−0.289 (0.4370)

Abbreviations: BMD = Bone Mineral Density; BMC = Bone Mineral Content; LMI = Lean Mass Index; FMI = Fat Mass Index. *N* = Total number of subjects in treatment group; *n* = Total number of non-missing subjects in specified category; SD = Standard Deviation; CFB = Change from baseline; LS Mean = Least squares mean. Note: The mean difference was estimated using an analysis of covariance adjusted for randomization stratification factors study center, gender, treatment × gender, age (numeric) and baseline value. * Results with *p*-values below 0.05 were deemed statistically significant. ^$^ Note: Total Body Z-scores were analyzed only for children aged ≥ 5 years, as validated DXA reference data are unavailable for younger ages, resulting in a reduced sample size for this analysis.

## Data Availability

The data presented in this study are available on request from the corresponding author.

## Abbreviations

The following abbreviations are used in this manuscript:

ANCOVA	Analyzed using analysis of covariance
BMC	Bone mineral content
BMD	Bone mineral density
BMI	Body Mass Index
CFB	Changes from baseline
CTRI	Clinical Trials Registry of India
DC	Dietary counselling
DXA	Dual-energy X-ray absorptiometry
FMI	Fat Mass Index
ICMR	Indian Council of Medical Research
LAR	Legal Authorized Representative
LMI	Lean Mass Index
ONS	Oral Nutritional Supplements
SPROUT	Supporting Pediatric GRowth and Health OUTcomes
WHO	World Health Organization
